# Peripheral blood lipid and liver and kidney function test results in long-term night shift nurses: a cross-sectional study in South China

**DOI:** 10.3389/fendo.2023.1237467

**Published:** 2023-10-11

**Authors:** Yang Zhao, Xunhao Lu, Yanghua Wang, Yiyi Cheng, Qiao He, Rongqi Qin, Wenrui Li, Haizhou Liu, Yuanfang Liu

**Affiliations:** ^1^ Department of Radiology, Guangxi Medical University Cancer Hospital, Nanning, China; ^2^ Department of Research, Guangxi Medical University Cancer Hospital, Nanning, China; ^3^ Department of Research, Guangxi Cancer Molecular Medicine Engineering Research Center, Nanning, China; ^4^ Department of Traditional Chinese Medicine, Guangxi Medical University Cancer Hospital, Nanning, China

**Keywords:** shift work (MeSH), nurses, liver function, kidney function, circadian rhythm

## Abstract

**Purpose:**

This study aimed to elucidate the effects of long-term day and night shifts on liver function and lipid metabolism in a group of nurses.

**Methods:**

This cross-sectional study in December 2019 was based on a group of nurses. A total of 1,253 physically healthy caregivers were included, including 1231 women and 22 men. A total of 886 nurses had long-term shift work (working in a rotating system for >1 year). The receiver operating characteristic (ROC) curve and logistic regression analyses were used to evaluate factors related to long-term shift work.

**Results:**

We observed differences in liver and kidney indicators between the non-night and night shift groups. The ROC curve revealed that CHO (AUC: 62.4%), LDLC (AUC: 62%), and GLUO (AUC: 61.5%) were more related to the night shift. Logistic regression analysis showed that night shift work was associated significantly with CREA (log (OR) = −0.02, 95% CI: −0.04 to −0.01), CHO (log (OR) = −0.38, 95% CI: −0.67 to −0.09), and GLUO (log (OR) = −0.35, 95% CI: −0.56 to −0.17). This correlation was observed only for CHO and LDHC (CHO: log (OR) = −0.55, 95% CI: −0.98 to −0.12; LDLC: log (OR) = 0.83, 95% CI: 0.32, 1.4) after age standardization. After using propensity score matching, we did not find evidence to support that the indicators differed between night and non-night shift groups.

**Conclusion:**

Our study observed an association of long-term night work with abnormal liver and kidney function and dyslipidemia, but the difference was not significant after strict age matching. Although these findings may support interventions for long-term night shift nurses, more detailed studies are needed to confirm.

## 1 Introduction

Circadian rhythms, observed in physiological functions in various species, are controlled by a master pacemaker in the brain called the suprachiasmatic nucleus. Circadian rhythm disruption significantly impacts people’s health and is associated with some diseases (1). At present, night shift work frequently occurs in contemporary society due to the increase in work pressure. Long-term night shift work will increase the chance of type 2 diabetes and the risk of cardiovascular disease (2). In addition, night shift work can also lead to a decrease in immunity and an increase in the risk of breast cancer. These are disadvantages for nurses, who are mainly female and often need to work night shift (3). Recent studies have also found that night shift work has a particular impact on liver and kidney function, such as the occurrence of non-alcoholic fatty liver disease and renal function decline (4, 5).

The changes in type 2 diabetes mellitus, cardiovascular disease, and liver and kidney function in the body are mainly manifested in blood biochemical indicators. Type 2 diabetes is characterized by elevated blood glucose, waist-to-hip ratio, body fat percentage, and visceral fat area (6). Cardiovascular diseases such as myocardial infarction may have elevated troponin and creatine kinase. The biochemical indicators related to liver and kidney function mainly include alanine aminotransferase (ALT), aspartate aminotransferase (AST), creatinine (CREA), uric acid (UA), and UREA nitrogen (UREA).

Studies have shown that night shift work significantly correlates with liver and kidney dysfunction (4, 7). Night shift work can affect liver function, leading to a condition known as non-alcoholic fatty liver disease (NAFLD) (8–10). The kidneys filter waste products from the blood and maintain fluid balance in the body. However, night shift work can affect kidney function, leading to a condition known as chronic kidney disease (CKD) (11–13). Night shift work can adversely affect liver and kidney function, leading to serious health problems (11). It is essential to minimize these effects, especially for the female nurse population.

Due to the lack of studies on the liver and kidney function and metabolic status of night shift nurses, the results of this study may provide some clues to the factors associated with abnormal indicators related to nurses. Through cross-sectional studies, we expected that night shift colleagues might have some abnormalities in liver and kidney function and metabolic status compared with their non-shift colleagues. Therefore, the main goal of this work was to examine the relationship between shift night work and liver and kidney function and metabolic status.

## 2 Method

### 2.1 Study population

**Figure 1:**
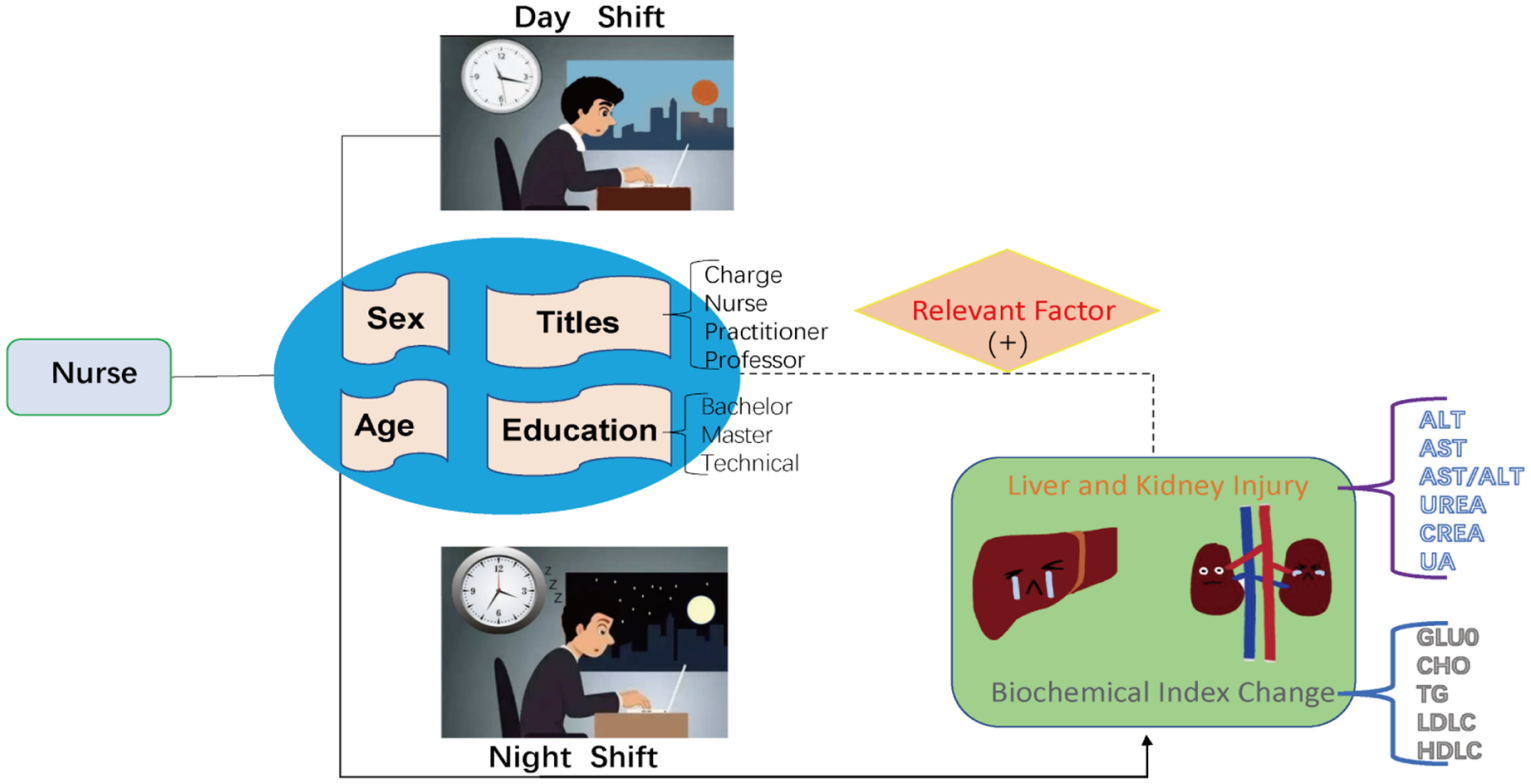
Working mode and Distribution of GLUO and other indicators in shift work nurses and normal population.

A total of 1,253 physically healthy caregivers were included after applying the condition (**Figure 1**), including 1,231 women and 22 men. A total of 886 nurses had long-term shift work (working in a rotating system for >1 year); the population’s average age was 30 years; 367 nurses without long-term shift work served as a control group, with an average age of 43 years. All nurses worked in hospitals in southern China in December 2019; therefore, the study sample included only nurses. The ethical approval number was LW2023089. Nurses who meet the following criteria were selected (1): healthy, physical examination results confirmed that the body is average; (2) no hypertension, hyperlipemia, hyperglycemia, and other diseases; (3) the clinical features, hematological indexes, and inflammatory biological indexes were complete. The exclusion criteria were as follows: (1) nurses with hepatic or renal dysfunction; (2) nurses with acute or chronic hematological diseases, severe systemic infections, or autoimmune diseases; (3) nurses with mental illness; (4) the clinical characteristics and indexes required by the study needed to be completed.

### 2.2 Serum and plasma markers

In this study, we collected peripheral blood data from 1,253 healthcare workers diagnosed as usual by physical examination. These include fasting blood glucose (GLUO), ALT, AST, UREA, CREA, UA, total cholesterol (CHO), triglyceride (TG), high-density lipoprotein (HDLC), and low-density lipoprotein (LDLC). Data were collected from November to December 2019. Nurses needed at least 12 h of fasting.

We collected 5 mL of peripheral venous blood from all participants on an empty stomach and left them for serum precipitation with a centrifugation radius of 8 cm, 3,500 r/min, and centrifugation for 10 min. ALT, AST, UREA, CREA, UA, CHO, TG, HDLC, and LDLC were measured using a Beckman Coulter automated biochemical analyzer (provided by Beckman Coulter and performed following the instructions).

### 2.3 Assessment of night shift work

In this study, the three-person two-shift system is mainly a rotating night shift working arrangement. Night shift work was an arrangement in which day and night shifts alternated continuously for over a year. Nurses who had not worked night shifts for more than 1 year and nurses who had been working were defined as non-night shift nurses. In the three groups of two changes, each group had one-night shift (18:00–08:00) followed by one day off. Data on night shift work were obtained by reconciliation with hospital work records.

### 2.4 Logistic regression

Logistic regression is a statistical method to analyze the relationship between a categorical dependent variable and one or more independent variables. It is widely used to estimate the probability of a certain event occurring. The logistic regression algorithm fits a logistic function to the data, which maps any input value to a probability between 0 and 1. The logistic function is defined as: p = \frac {1} {1 + e^{−z}}. where “p” is the predicted probability, “z” is the weighted sum of the input features, and “e” is the mathematical constant of approximately 2.71828.

### 2.5 Statistical analysis

The characteristics of health nurses working long shifts were summarized and described. Frequency distributions represent categorical variables, whereas continuous variables are reported by median and interquartile range. Markers with disease and clinicopathological features were explored by the Wilcoxon rank-sum test or Kruskal–Wallis test. The receiver operating characteristic (ROC) curve was established to select these continuous indicators’ optimal threshold and diagnostic accuracy. A convincing shift nurse model was based on multivariate logistic regression analysis. Propensity score matching (14) (PSM) is a statistical method used to assess the disposition effect. Broadly speaking, it classifies samples according to their characteristics, and the differences between different classes of samples can be considered as unbiased estimates of the disposition effect. R-4.22 software was used for statistical analysis.

## 3 Result

**Table 1**; **Figure 2** show the sociodemographic characteristics of nurses according to night shift work status. Work status significantly affects age, liver function, and lipid metabolism. Among 1,253 subjects, The median (upper quartile–lower quartile) of GLUO, ALT, AST, AST/ALT, UREA, CREA, CHO, TG, and LDLC in 886-night shift work nurses was 4.71 (4.39, 5.03), 16 (13, 23), 24 (21, 28), 1.47 (1.13, 1.75), 4.60 (3.80, 5.40), 60 (54, 68), 5.38 (4.80, 6.07), 1.01 (0.72, 1.49), and 3.26 (2.71, 3.79) U/L, respectively. In addition, among the subjects, 367 (29.3%) had never worked the night shift. The levels of GLUO, ALT, AST, AST/ALT, UREA, CREA, CHO, TG, and LDLC are 4.53 (4.22, 4.79), 14 (12, 19), 23 (20, 26), 1.57 (1.24, 1.85), 4.30 (3.60, 5.10), 57 (51, 64), 5.00 (4.39, 5.57), 0.87 (0.63, 1.25), and 2.87 (2.40, 3.42) which differed by work status, and lower levels of GLUO, ALT, AST, UREA, CREA, CHO, TG, and ALP and higher ALT/AST and LDLC were found among night shift work nurses. The proportion of males engaged in night shift work was not significantly higher than that of women (Supplementary Information, **Table S1**), and the night shift rate varied between educational levels; their blood test indicators also differed significantly (Supplementary Information, **Table S2**). In addition, also job title is a factor that affects the night shift work rate, and blood test indicators differ for nurses with different titles (Supplementary Information, **Table S3**).

**Table 1:** Characteristics of study participants.

Characteristic	No, N = 367^1^	Yes, N = 886^1^	p-value^2^
Sex			0.5
Female	359 (98%)	872 (98%)	
Male	8 (2.2%)	14 (1.6%)	
Education			0.069
Bachelor	5 (1.4%)	3 (0.3%)	
Master	0 (0%)	3 (0.3%)	
Technical	362 (99%)	880 (99%)	
Titles			<0.001
Charge	175 (48%)	222 (25%)	
Nurse	11 (3.0%)	123 (14%)	
Practitioner	59 (16%)	538 (61%)	
Professor	122 (33%)	3 (0.3%)	
Age	43 (37, 52)	30 (28, 33)	<0.001
GLUO	4.71 (4.39, 5.03)	4.53 (4.22, 4.79)	<0.001
ALT	16 (13, 23)	14 (12, 19)	<0.001
AST	24 (21, 28)	23 (20, 26)	<0.001
AST/ALT	1.47 (1.13, 1.75)	1.57 (1.24, 1.85)	<0.001
UREA	4.60 (3.80, 5.40)	4.30 (3.60, 5.10)	<0.001
CREA	60 (54, 68)	57 (51, 64)	<0.001
UA	277 (230, 343)	268 (231, 318)	0.025
CHO	5.38 (4.80, 6.07)	5.00 (4.39, 5.57)	<0.001
TG	1.01 (0.72, 1.49)	0.87 (0.63, 1.25)	<0.001
HDLC	1.47 (1.30, 1.76)	1.49 (1.28, 1.71)	0.7
LDLC	3.26 (2.71, 3.79)	2.87 (2.40, 3.42)	<0.001

^1^n (%); median (IQR)

^2^Pearson’s chi-squared test; Fisher’s exact test; Wilcoxon rank-sum test

NO, no overnight;YES, overnight;GLUO, fasting blood sugar; ALT, alanine transaminase; AST, aspartate transaminase; UREA, urea; CREA, creatinine; UA, uric acid; CHO, total cholesterol; TG, triglycerides; HDLC, high-density lipoprotein cholesterol; LDLC, low-density lipoprotein cholesterol.

**Figure 2:**
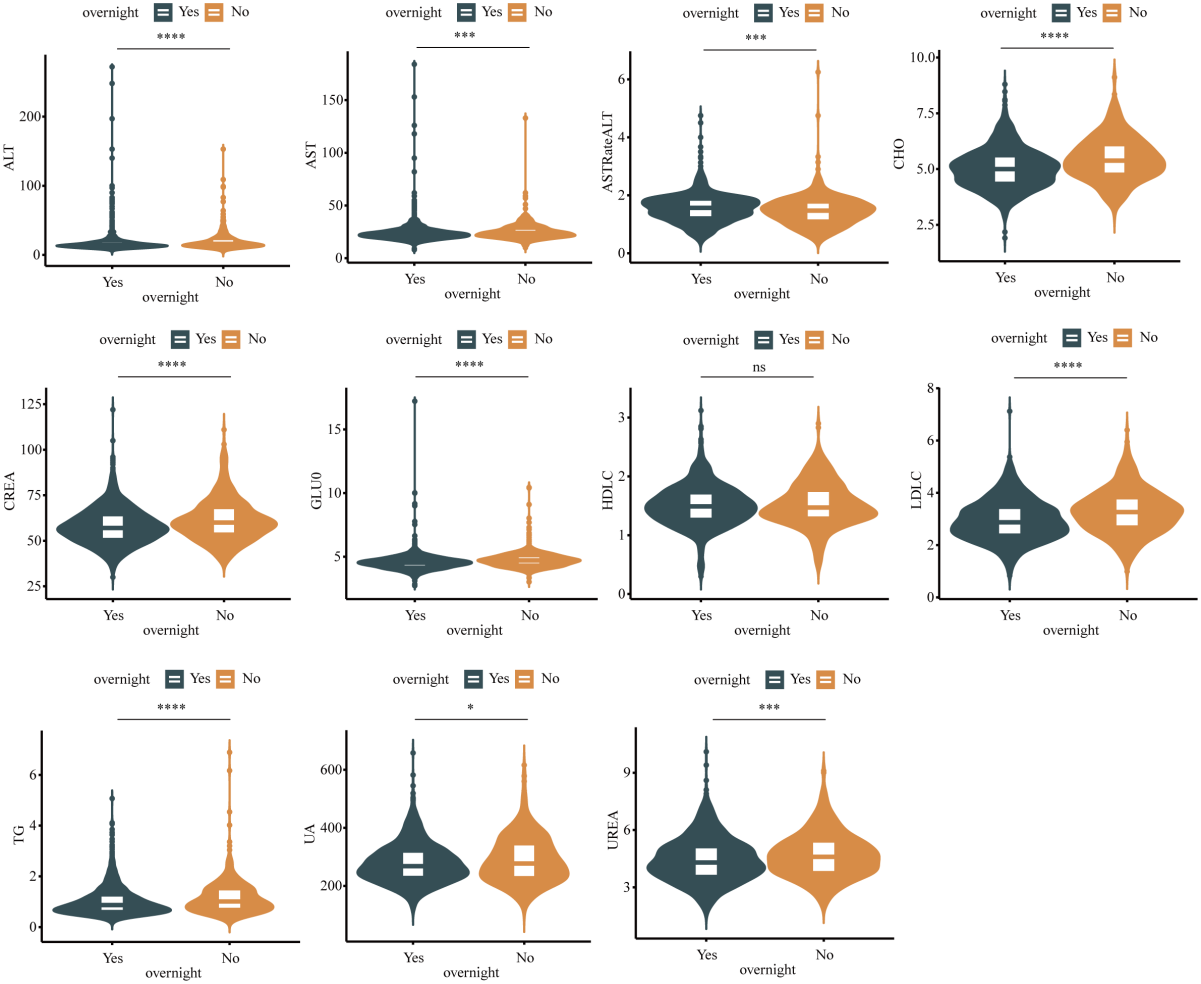
Boxplot in shift work nurses and normal population. Differences between groups estimated by Mann–Whitney U as appropriate. (*P <0.05, ***P <0.001, ****P<0.0001, ns P>0.05).

**Figure 3:**
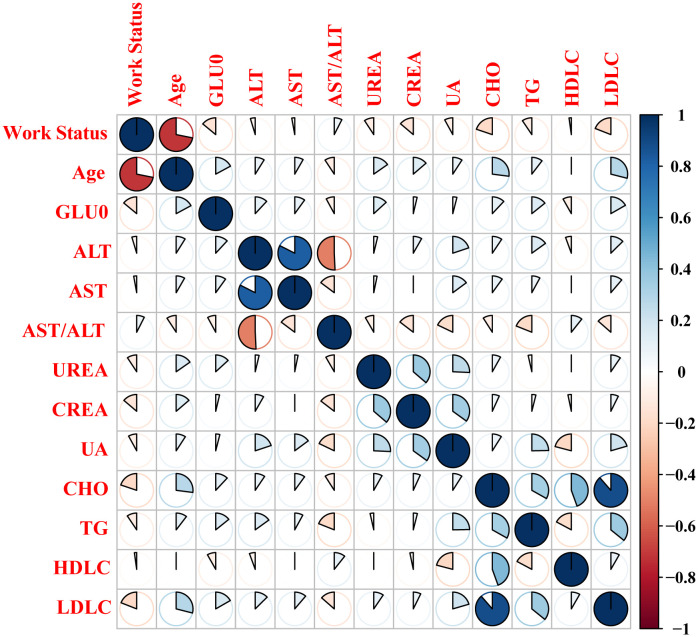
Visualization of the correlation matrix between biochemical indicators and Working Status.

Correlation heat maps were used to analyze the relationship between work status, liver and kidney function, and lipid metabolism. Correlation coefficient heat maps were obtained based on Pearson correlation at unadjusted age. Correlations were found between all indicators of liver and kidney function, lipid metabolism, and night shift work status (**Figure 3**). Age, GLUO, UREA, CREA, CHO, TG, and LDLC had higher correlations. The study showed that the relationship between different liver and kidney functions, lipid metabolism indicators, and night shift work status was attenuated before and after adjustment for age and gender (Supplementary Information, **Figure S1**). Although more frequent shift work exposure increased the odds of elevated AST, AST/ALT, and LDLC compared with those nurses who never worked night shifts, this association was not statistically significant.

**Figure 4:**
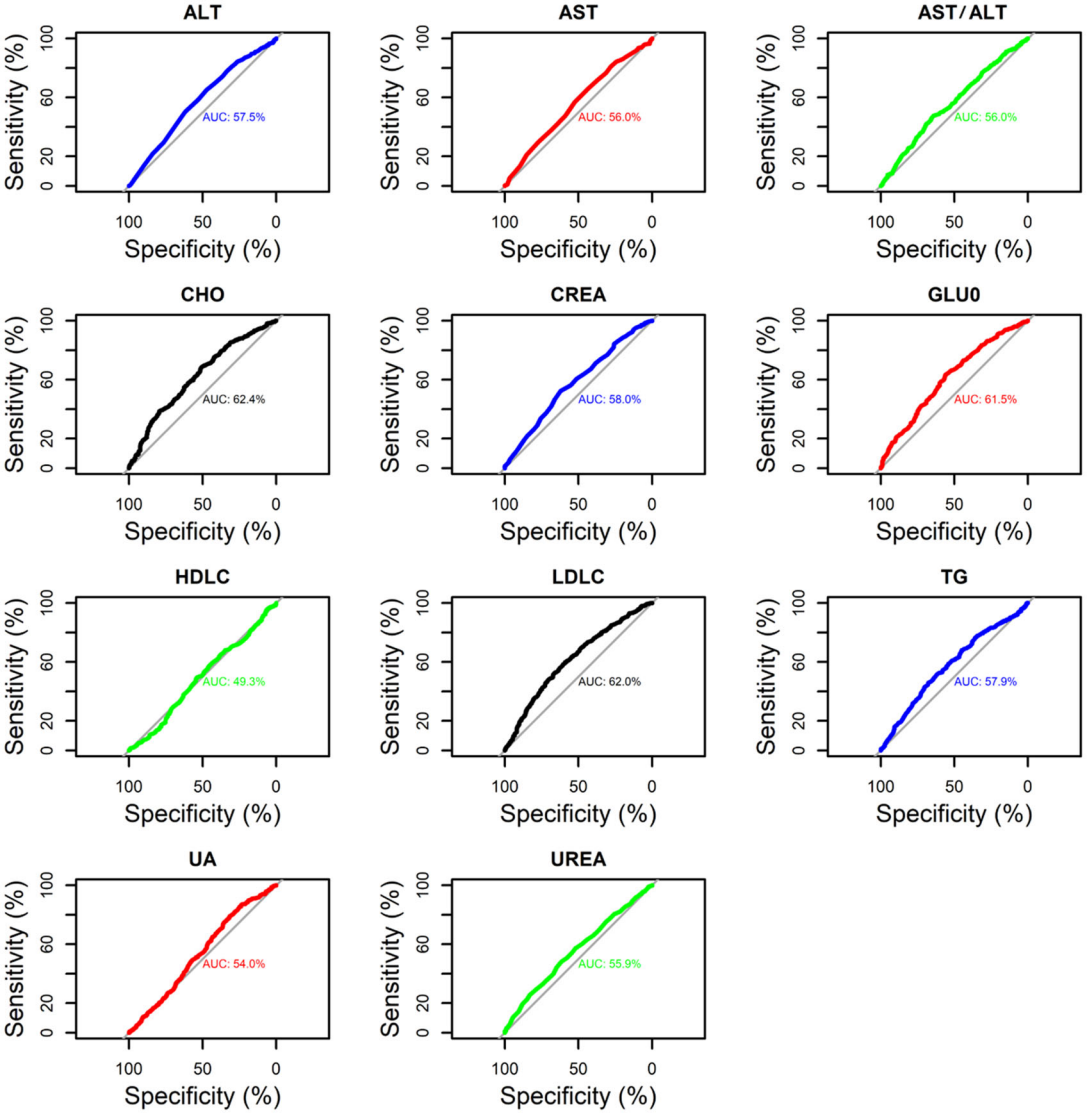
ROC curve analysis of the relationship between the presence or absence of night shifts and liver and kidney function and lipid indices.

The ROC curve analyzes the optimal thresholds and their sensitivity and specificity drawn in **Figure 4**. The area under the ROC curve reflects the accuracy of the diagnostic test. From this analysis and evaluation, we can find that the change of rhythm in the night shift population will lead to changes in GLUO, AST, ALT, AST/ALT, UREA, CREA, CHO, TG, LDLC, and HLDC indicators, which will lead to changes in our entire liver and kidney function indicators, of which CHO, LDLC, and GLUO change the most. The diagnostic value was more significant than 0.6. It was shown that night shifts leading to circadian rhythm changes were the most correlated with CHO, LDLC, and GLUO. Therefore, we intend to use machine learning and regression analysis methods for subsequent analysis.

**Figure 5:**
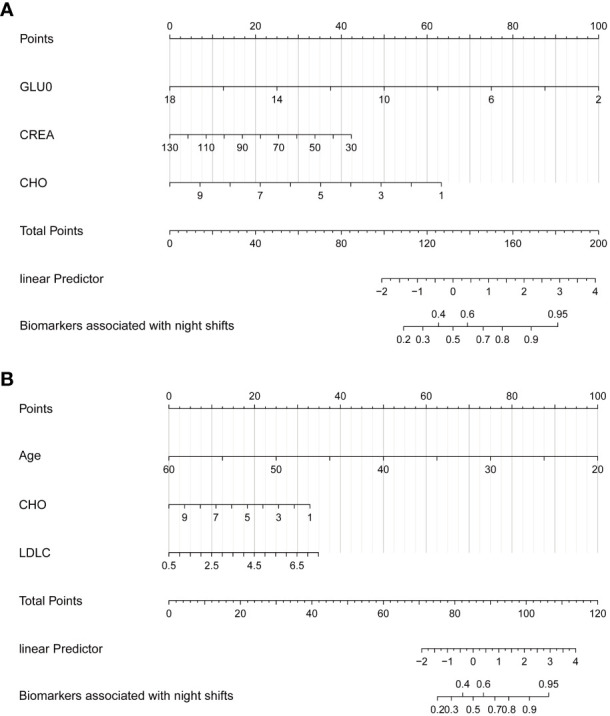
Nomogram demonstrates the relationship between long-term night shift work and liver and kidney function and lipid metabolism. **(A)** General Model. **(B)** Age-adjusted model.

**Table 2** summarizes the results of a one-way logistic analysis of the relationship between long-term night work and liver and kidney function and lipids. The results show that univariate analysis of the association between long-term night work and changes in liver and kidney function indicators (AST/ALT, CHO, CREA, GLUO) was statistically significant. Significance was as follows: AST/ALT—OR = 1.40, 95% CI: 1.10–1.81; CHO—OR = 0.63, 95% CI: 0.55–0.72; CREA—OR = 0.97, 95% CI: 0.96–0.98); GLUO—OR = 0.65, 95% CI: 0.53–0.78; UREA—OR = 0.84, 95% CI: 0.76–0.93; UA—OR = 1.00, 95% CI: 1.00–1.00. The association between long-term night work and changes in lipid markers (LDLC, TG) was statistically significant. Significance was as follows: LDLC—OR = 0.60, 95% CI: 0.51–0.70; TG—OR = 0.74, 95% CI: 0.62–0.89. In contrast, **Table 3** shows the multifactorial logistic analysis of the relationship between work, liver, kidney function, and lipids at night. Only work status was observed to be associated with CREA, GLUO, and CHO (CREA: log(OR) = −0.02, 95% CI: −0.04 to −0.01; CHO: log(OR) = −0.38, 95% CI: −0.67 to −0.09; GLUO: log(OR) = −0.35, 95% CI: −0.56 to −0.17). However, after age standardization, it was associated with only CHO and LDHC with work status (CHO: log(OR) = −0.55, 95% CI: −0.98 to −0.12; LDLC: log(OR) = 0.83, 95% CI: 0.32, 1.4). Based on multifactorial regression, we constructed nomograms to predict the relationship between the biomarkers and the working status (**Figure 5**). **Figure 5A** shows that GLUO, CREA, and CHO were strongly associated with nurses’ night shift work status. In contrast, after age standardization (see **Figure 5B**), two lipid indicators, CHO and LDLC, were associated with the night shift work status of the age-standardized nurse group.

**Table 2:** Odds ratio for liver and kidney function and lipid metabolism based on univariate logistic regression.

Characteristic	N	Event N	OR^1^	95% CI^1^	p-value	q-value^2^
Age	1,253	886	0.71	0.68, 0.74	<0.001	<0.001
ALT	1,253	886	1.0	0.99, 1.00	0.11	0.13
AST	1,253	886	1.0	0.98, 1.01	0.32	0.35
AST/ALT	1,253	886	1.40	1.10, 1.81	0.007	0.009
CHO	1,253	886	0.63	0.55, 0.72	<0.001	<0.001
CREA	1,253	886	0.97	0.96, 0.98	<0.001	<0.001
GLUO	1,253	886	0.65	0.53, 0.78	<0.001	<0.001
HDLC	1,253	886	0.88	0.63, 1.22	0.43	0.43
LDLC	1,253	886	0.60	0.51, 0.70	<0.001	<0.001
TG	1,253	886	0.74	0.62, 0.89	0.001	0.002
UA	1,253	886	1.00	1.00, 1.00	0.005	0.007
UREA	1,253	886	0.84	0.76, 0.93	0.001	0.002

^1^OR, odds ratio; CI, confidence Interval.

^2^False discovery rate correction for multiple testing.

GLUO, fasting blood sugar; ALT, alanine transaminase; AST, aspartate transaminase; UREA, urea; CREA, creatinine; UA, uric acid; CHO, total cholesterol; TG, triglycerides; LDLC, low-density lipoprotein cholesterol.

**Table 3:** Odds ratio for liver and kidney function and lipid metabolism based on multivariable logistic regression.

Characteristic	Log(OR)^1^	Model 1*		Log(OR)^1^	Model 2**	
		95% CI^1^	p-value		95% CI^1^	p-value
GLUO	−0.35	−0.56, −0.17	<0.001	−0.13	−0.34, 0.10	0.2
AST/ALT	0.13	−0.12, 0.39	0.3	0.03	−0.34, 0.41	0.9
UREA	−0.05	−0.16, 0.07	0.4	0.13	−0.04, 0.30	0.15
CREA	−0.02	−0.04, −0.01	<0.001	−0.02	−0.04, 0.00	0.061
UA	0.00	0.00, 0.00	0.8	0.00	0.00, 0.00	0.4
CHO	−0.38	−0.67, −0.09	0.010	−0.55	−0.98, −0.12	0.011
TG	−0.03	−0.24, 0.18	0.8	−0.16	−0.46, 0.14	0.3
LDLC	−0.04	−0.38, 0.30	0.8	0.83	0.32, 1.4	0.002

^1^OR, odds ratio; CI, confidence interval.

*Multivariable models.

**Multivariable models adjusted for age.

GLUO, fasting blood sugar; ALT, alanine transaminase; AST, aspartate transaminase; UREA, urea; CREA, creatinine; UA, uric acid; CHO, total cholesterol; TG, triglycerides; LDLC, low-density lipoprotein cholesterol.

Finally, we used propensity score matching, PSM, a statistical method used to assess disposition effects, to match permanent night shift nurses to non-night shift nurses on a 1:2 basis, such that clinical characteristics (e.g., age, gender) should all be substantially similar. However, comparing the two groups after matching showed no significant differences in all indicators, as shown in Supplementary Information, **Table S4**.

## 4 Discussion

With the progress of research on circadian rhythm, it has been found that circadian rhythm disorder is related to many diseases. People who work shifts experience disrupted sleep, gastrointestinal disorders, general malaise, and an increased risk of cancer and heart disease. The impact of the circadian system on immune disorders, mood disorders, neuropsychiatric disorders, aging, renal disease, and cancer has been reviewed by others (15). Long-term night shift work may reduce the level of melatonin for a long time, and night shift work can affect the level and time of melatonin production. Studies have shown that melatonin significantly impacts sleep quality, and insufficient melatonin will affect sleep quality, which may be one of the reasons for sleep disruption in shift workers (16, 17). Compared with the day shift workers, abdominal pain, diarrhea, constipation, and change in bowel habits are more common in shift workers, and the incidence of gastric ulcers and small intestinal ulcers is higher. These symptoms may indicate circadian rhythm disorder in shift workers (18, 19). After the occurrence of cancer, various tumor markers can be used as an evaluation index of circadian disruption. Heart rate variability (HRV) during sleep is significantly different from that of day shift workers in male shift workers but not in female shift workers, which can be used as an indicator to evaluate the risk of cardiovascular disease in male shift workers (20).

Our study examined a cohort of 1,253 healthcare workers, divided all participants into two groups based on whether they had long shift work experience, and collected and investigated associations between peripheral blood ALT, AST, ALT/AST, UREA, CREA, UA, CHO, TG, HDL-C, LDL-C, and other traditional markers. In univariate analysis, CHO, LDL-C, CREA, and GLUO significantly differed between the long-term shift and control populations. Only CREA and GLUO are considered meaningful biochemical indicators in multivariate analysis. Although many previous studies have suggested that circadian rhythm disturbances are associated with chronic liver and kidney disease, the results needed to be consistent. Ghiasvand et al. studied 424 railway workers aged 21 and 64 (21). The parametric test showed no difference in GLUO levels between shift and day workers. In informal male workers with insufficient sleep time and shift work, multivariate logistic regression analysis found that elevated GLUO levels were associated with shift work (22). In a study of the association between shift work and chronic kidney disease in manual workers, the change in the ratio of CREA to urine protein was not significant in the male worker group. Still, it significantly changed in the female worker group (odds ratio = 2.34, 95% confidence interval = 1.35, 4.07) (23).

After adjusting for age, we found that prolonged night work increased nurses’ risk of dyslipidemia and abnormal liver and kidney function. Humans eat, metabolize nutrients, and sleep according to circadian rhythms controlled by the biological clock (24, 25). It has been suggested that disruption of the biological clock associated with night work may lead to obesity, impaired insulin secretion, and abnormal glucose homeostasis (24, 26). Notably, the overlap has been observed between mechanisms associated with insulin resistance and atherosclerosis (a consequence of dyslipidemia), including elevated levels of glucose and free acid that cause oxidative stress, activation of pro-inflammatory pathways, low levels of HDL, and high levels of triglycerides (27, 28). The biological clock regulates lipid metabolism, and it has been suggested that periodic disruptions in circadian rhythms negatively affect lipid metabolism (29–32). Therefore, long-term shift night work is more strongly associated with dyslipidemia. Still, in the present study, although alterations in lipids were observed in shift nurses, we only observed an increase in LDLC in nurses due to night work after age-propensity matching. Still, the difference between the two groups was not significant.

This study describes the effects on lipid metabolism biomarkers and renal function. Since it is a cross-sectional study lacking follow-up observations, we will add follow-up for different diseases in subsequent studies based on the existing cohort. During the initial design of the cohort study, urine sampling of the cohort population was missing because the study was designed only for peripheral blood indicators, which led to the study of renal function being incomplete in this study, and we will improve the relevant tests in the follow-up.

Among the nurses who participated in this experiment, disease screening and other interference factors were not considered when checking the indicators, which may have a specific impact on the experimental data. This experiment mainly reflects the function of the organs or systems of the body through the blood biochemical indicators. Night shift’s influence on the body’s function needs to be more comprehensive and intuitive. Further changes in liver and kidney function and lipid indices may be due to differences in age composition between subgroups. In a similar study, we also found that age was strongly associated with night shift schedule, with senior nurses tending not to schedule or to schedule fewer night shifts, which also creates an error in the study, as several studies have concluded that dyslipidemia and age are closely related. After age-based propensity matching in our research, such differences disappeared, showing, on the one hand, that night shift scheduling is associated with liver function and lipid metabolism and, on the other hand, that such differences may have a limited effect on liver and kidney function and lipids compared with age in the nurse population. Behavioral interventions for night shifts in groups of nurses are feasible, but their benefits need not be overstated.

## 5 Conclusions

The rhythm disorder caused by shift work easily causes various diseases, including cardiovascular, and neuropsychiatric, digestive system diseases, and even cancer. In this study, we confirmed the effect of rhythm disorder on liver and kidney function and lipid metabolism. Our experiment found that GLUO, CHO, TG, LDL-C, HDL-C, and other indicators also have clinical significance, which helps to confirm that the occurrence of cardiovascular disease and type 2 diabetes mellitus is significantly related to rhythm disorder. It is also helpful for the prevention of clinical conditions. We hope this study can remind the nurse community of the harm caused by circadian disturbance to the body and prevent the occurrence and development of the resulting diseases.

## Data availability statement

The original contributions presented in the study are included in the article/**Supplementary Material**. Further inquiries can be directed to the corresponding authors.

## Ethics statement

The studies involving humans were approved by Guangxi Medical University Cancer Hospital Ethics Committee. The studies were conducted in accordance with the local legislation and institutional requirements. Written informed consent for participation in this study was provided by the participants’ legal guardians/next of kin.

## Author contributions

YL and HL designed and conceived the study. YC, QH, and RQ conducted the research study. HL provided the experimental resources. YL, WL, and QH conducted the experiments, RQ and WL collected and managed the data. XL, YW, and HL analyzed the data formally. XL, YC, and HL performed the data analysis for validation. HL, XL, YW, YL, and YC visualized and presented the data, XL, YW, and YL wrote this manuscript. HL reviewed and edited the manuscript. All authors contributed to the article and approved the submitted version.
