# Supplementary material for: Peripheral blood lipid and liver and kidney function test results in long-term night shift nurses: a cross-sectional study in South China
Source: Front Endocrinol (Lausanne). 2023 Oct 11;14:1237467. doi: 10.3389/fendo.2023.1237467 (PMC10613520; doi:10.3389/fendo.2023.1237467)
Supplement: Supplementary file 1 [file DataSheet_1.zip › Supplementary/Table S1.docx]

| Characteristic | Female, N = 1,231^1^ | Male, N = 22^1^ | p-value^2^ |
| --- | --- | --- | --- |
| **Education** |  |  | >0.9 |
| Bachelor | 8 (0.6%) | 0 (0%) |  |
| Master | 3 (0.2%) | 0 (0%) |  |
| Technical | 1,220 (99%) | 22 (100%) |  |
| **overnight** | 872 (71%) | 14 (64%) | 0.5 |
| **Titles** |  |  | <0.001 |
| charge | 388 (32%) | 9 (41%) |  |
| Nurse | 124 (10%) | 10 (45%) |  |
| Practitioner | 594 (48%) | 3 (14%) |  |
| Professor | 125 (10%) | 0 (0%) |  |
| **Age** | 32 (29, 38) | 34 (28, 37) | >0.9 |
| **GLU0** | 4.56 (4.26, 4.87) | 4.59 (4.30, 4.88) | >0.9 |
| **ALT** | 15 (12, 20) | 15 (12, 28) | 0.4 |
| **AST** | 23 (20, 27) | 24 (20, 29) | 0.4 |
| **AST/ALT** | 1.53 (1.21, 1.82) | 1.41 (0.93, 1.86) | 0.5 |
| **UREA** | 4.40 (3.70, 5.20) | 4.65 (3.97, 5.55) | 0.2 |
| **CREA** | 58 (52, 64) | 69 (60, 79) | <0.001 |
| **UA** | 269 (230, 324) | 370 (311, 396) | <0.001 |
| **CHO** | 5.11 (4.50, 5.72) | 5.16 (4.09, 5.54) | 0.7 |
| **TG** | 0.91 (0.65, 1.33) | 1.04 (0.82, 1.56) | 0.11 |
| **HDLC** | 1.48 (1.29, 1.73) | 1.45 (1.10, 1.59) | 0.13 |
| **LDLC** | 2.98 (2.46, 3.51) | 3.50 (2.43, 3.94) | 0.3 |
| ^1^n (%); Median (IQR) | | | |
| ^2^Fisher's exact test; Pearson's Chi-squared test; Wilcoxon rank sum test | | | |
