# Supplementary material for: Peripheral blood lipid and liver and kidney function test results in long-term night shift nurses: a cross-sectional study in South China
Source: Front Endocrinol (Lausanne). 2023 Oct 11;14:1237467. doi: 10.3389/fendo.2023.1237467 (PMC10613520; doi:10.3389/fendo.2023.1237467)
Supplement: Supplementary file 1 [file DataSheet_1.zip › Supplementary/Table S4.docx]

**Table S4 Analysis after using propensity matching Cohort baseline data disaggregated by Day and night shifts work Status.**

| **Characteristic** | No, N = 135^1^ | Yes, N = 234^1^ | **p-value**^2^ |
| --- | --- | --- | --- |
| **Sex** |  |  | 0.5 |
| Female | 130 (96%) | 228 (97%) |  |
| Male | 5 (3.7%) | 6 (2.6%) |  |
| **Education** |  |  | 0.11 |
| Bachelor | 5 (3.7%) | 2 (0.9%) |  |
| Master | 0 (0%) | 3 (1.3%) |  |
| Technical | 130 (96%) | 229 (98%) |  |
| **Titles** |  |  | <0.001 |
| Charge | 60 (44%) | 102 (44%) |  |
| Nurse | 11 (8.1%) | 28 (12%) |  |
| Practitioner | 49 (36%) | 101 (43%) |  |
| Professor | 15 (11%) | 3 (1.3%) |  |
| **Age** | 35 (32, 38) | 34 (32, 37) | 0.080 |
| **GLU0** | 4.62 (4.26, 4.96) | 4.53 (4.23, 4.79) | 0.072 |
| **ALT** | 15 (12, 20) | 14 (12, 19) | 0.2 |
| **AST** | 23 (20, 26) | 23 (20, 26) | >0.9 |
| **AST/ALT** | 1.52 (1.20, 1.76) | 1.62 (1.26, 1.86) | 0.085 |
| **UREA** | 4.40 (3.70, 5.30) | 4.40 (3.70, 5.30) | >0.9 |
| **CREA** | 59 (52, 64) | 57 (52, 63) | 0.2 |
| **UA** | 276 (230, 330) | 273 (232, 325) | 0.9 |
| **CHO** | 5.00 (4.43, 5.47) | 5.04 (4.53, 5.52) | 0.5 |
| **TG** | 0.90 (0.67, 1.48) | 0.89 (0.67, 1.32) | 0.7 |
| **HDLC** | 1.46 (1.29, 1.68) | 1.45 (1.28, 1.65) | 0.7 |
| **LDLC** | 2.89 (2.38, 3.44) | 2.99 (2.50, 3.56) | 0.2 |
| ^1^n (%); Median (IQR) | | | |
| ^2^Fisher's exact test; Pearson's Chi-squared test; Wilcoxon rank sum test | | | |
