# Supplementary material for: Peripheral blood lipid and liver and kidney function test results in long-term night shift nurses: a cross-sectional study in South China
Source: Front Endocrinol (Lausanne). 2023 Oct 11;14:1237467. doi: 10.3389/fendo.2023.1237467 (PMC10613520; doi:10.3389/fendo.2023.1237467)
Supplement: Supplementary file 1 [file DataSheet_1.zip › Supplementary/Table S3.docx]

| Characteristic | charge, N = 397^1^ | Nurse, N = 134^1^ | Practitioner, N = 597^1^ | Professor, N = 125^1^ | p-value^2^ |
| --- | --- | --- | --- | --- | --- |
| **Sex** |  |  |  |  | <0.001 |
| Female | 388 (98%) | 124 (93%) | 594 (99%) | 125 (100%) |  |
| Male | 9 (2.3%) | 10 (7.5%) | 3 (0.5%) | 0 (0%) |  |
| **Education** |  |  |  |  | 0.012 |
| Bachelor | 4 (1.0%) | 0 (0%) | 4 (0.7%) | 0 (0%) |  |
| Master | 0 (0%) | 0 (0%) | 0 (0%) | 3 (2.4%) |  |
| Technical | 393 (99%) | 134 (100%) | 593 (99%) | 122 (98%) |  |
| **overnight** | 222 (56%) | 123 (92%) | 538 (90%) | 3 (2.4%) | <0.001 |
| **Age** | 36 (33, 42) | 27 (25, 30) | 30 (28, 32) | 52 (45, 55) | <0.001 |
| **GLU0** | 4.63 (4.25, 4.92) | 4.50 (4.17, 4.74) | 4.52 (4.25, 4.81) | 4.72 (4.48, 5.07) | <0.001 |
| **ALT** | 15 (12, 20) | 15 (13, 20) | 15 (12, 19) | 17 (13, 23) | 0.010 |
| **AST** | 24 (20, 27) | 23 (20, 26) | 23 (20, 26) | 24 (21, 29) | 0.002 |
| **AST/ALT** | 1.53 (1.18, 1.82) | 1.56 (1.24, 1.84) | 1.54 (1.24, 1.83) | 1.47 (1.17, 1.79) | 0.3 |
| **UREA** | 4.40 (3.70, 5.10) | 4.10 (3.60, 5.10) | 4.40 (3.60, 5.20) | 4.80 (4.10, 5.60) | <0.001 |
| **CREA** | 59 (53, 65) | 57 (50, 64) | 57 (51, 63) | 59 (53, 68) | 0.004 |
| **UA** | 268 (232, 323) | 298 (238, 348) | 263 (225, 311) | 279 (236, 344) | <0.001 |
| **CHO** | 5.26 (4.67, 5.86) | 4.61 (4.08, 5.26) | 5.02 (4.42, 5.59) | 5.50 (5.00, 6.16) | <0.001 |
| **TG** | 0.92 (0.68, 1.35) | 0.90 (0.64, 1.26) | 0.84 (0.62, 1.25) | 1.16 (0.84, 1.56) | <0.001 |
| **HDLC** | 1.46 (1.30, 1.69) | 1.39 (1.22, 1.56) | 1.54 (1.29, 1.76) | 1.44 (1.25, 1.70) | <0.001 |
| **LDLC** | 3.16 (2.62, 3.66) | 2.64 (2.19, 3.50) | 2.83 (2.40, 3.34) | 3.48 (2.98, 3.93) | <0.001 |
| ^1^n (%); Median (IQR) | | | | | |
| ^2^Fisher's exact test; Pearson's Chi-squared test; Kruskal-Wallis rank sum test | | | | | |
