# Supplementary material for: Peripheral blood lipid and liver and kidney function test results in long-term night shift nurses: a cross-sectional study in South China
Source: Front Endocrinol (Lausanne). 2023 Oct 11;14:1237467. doi: 10.3389/fendo.2023.1237467 (PMC10613520; doi:10.3389/fendo.2023.1237467)
Supplement: Supplementary file 1 [file DataSheet_1.zip › Supplementary/Table S2.docx]

| Characteristic | Bachelor, N = 8^1^ | Master, N = 3^1^ | Technical, N = 1,242^1^ | p-value^2^ |
| --- | --- | --- | --- | --- |
| **Sex** |  |  |  | >0.9 |
| Female | 8 (100%) | 3 (100%) | 1,220 (98%) |  |
| Male | 0 (0%) | 0 (0%) | 22 (1.8%) |  |
| **overnight** | 3 (38%) | 3 (100%) | 880 (71%) | 0.069 |
| **Titles** |  |  |  | 0.012 |
| charge | 4 (50%) | 0 (0%) | 393 (32%) |  |
| Nurse | 0 (0%) | 0 (0%) | 134 (11%) |  |
| Practitioner | 4 (50%) | 0 (0%) | 593 (48%) |  |
| Professor | 0 (0%) | 3 (100%) | 122 (9.8%) |  |
| **Age** | 34 (32, 36) | 39 (38, 40) | 32 (29, 38) | 0.2 |
| **GLU0** | 4.87 (4.61, 5.20) | 4.43 (4.38, 4.80) | 4.56 (4.26, 4.87) | 0.2 |
| **ALT** | 14 (13, 15) | 11 (10, 13) | 15 (12, 20) | 0.2 |
| **AST** | 23 (22, 24) | 21 (21, 22) | 23 (20, 27) | 0.7 |
| **AST/ALT** | 1.64 (1.51, 1.84) | 1.91 (1.66, 2.11) | 1.53 (1.20, 1.82) | 0.2 |
| **UREA** | 5.30 (3.98, 5.58) | 6.10 (5.45, 6.50) | 4.40 (3.70, 5.20) | 0.064 |
| **CREA** | 59 (54, 64) | 63 (61, 64) | 58 (52, 65) | 0.5 |
| **UA** | 216 (211, 254) | 280 (257, 282) | 271 (231, 325) | 0.10 |
| **CHO** | 4.44 (4.00, 4.93) | 4.88 (4.80, 5.18) | 5.12 (4.50, 5.72) | 0.2 |
| **TG** | 0.75 (0.69, 1.09) | 1.31 (1.28, 1.37) | 0.91 (0.65, 1.33) | 0.3 |
| **HDLC** | 1.32 (1.04, 1.66) | 1.15 (1.13, 1.19) | 1.48 (1.29, 1.73) | 0.040 |
| **LDLC** | 2.17 (2.00, 2.69) | 3.14 (3.13, 3.46) | 2.98 (2.46, 3.53) | 0.10 |
| ^1^n (%); Median (IQR) | | | | |
| ^2^Fisher's exact test; Kruskal-Wallis rank sum test | | | | |
