# Supplementary figures and images for: Peripheral blood lipid and liver and kidney function test results in long-term night shift nurses: a cross-sectional study in South China
Source: Front Endocrinol (Lausanne). 2023 Oct 11;14:1237467. doi: 10.3389/fendo.2023.1237467 (PMC10613520; doi:10.3389/fendo.2023.1237467)

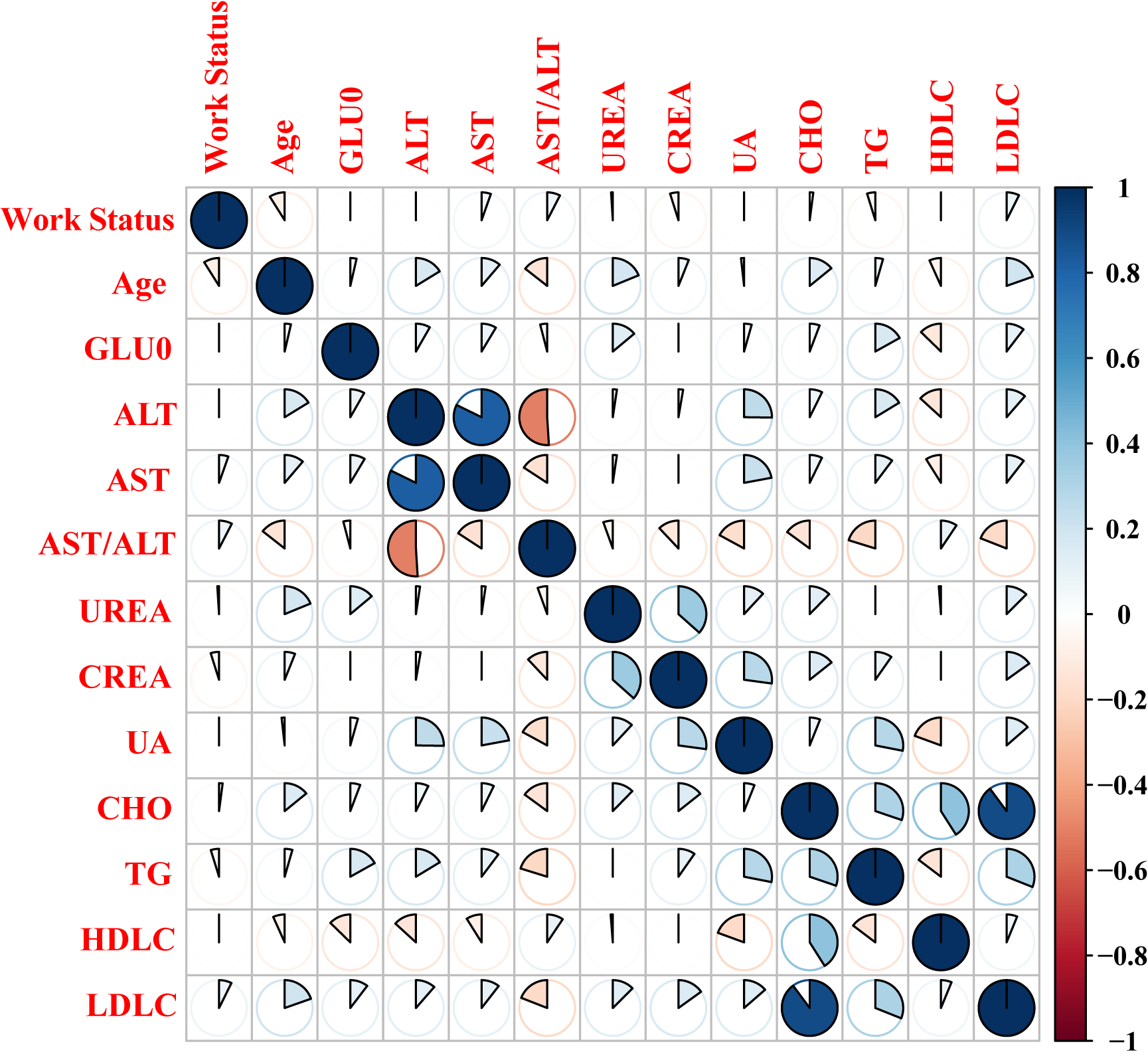

Supplement: Supplementary file 1 [file DataSheet_1.zip › Supplementary/Figue s1.tif]

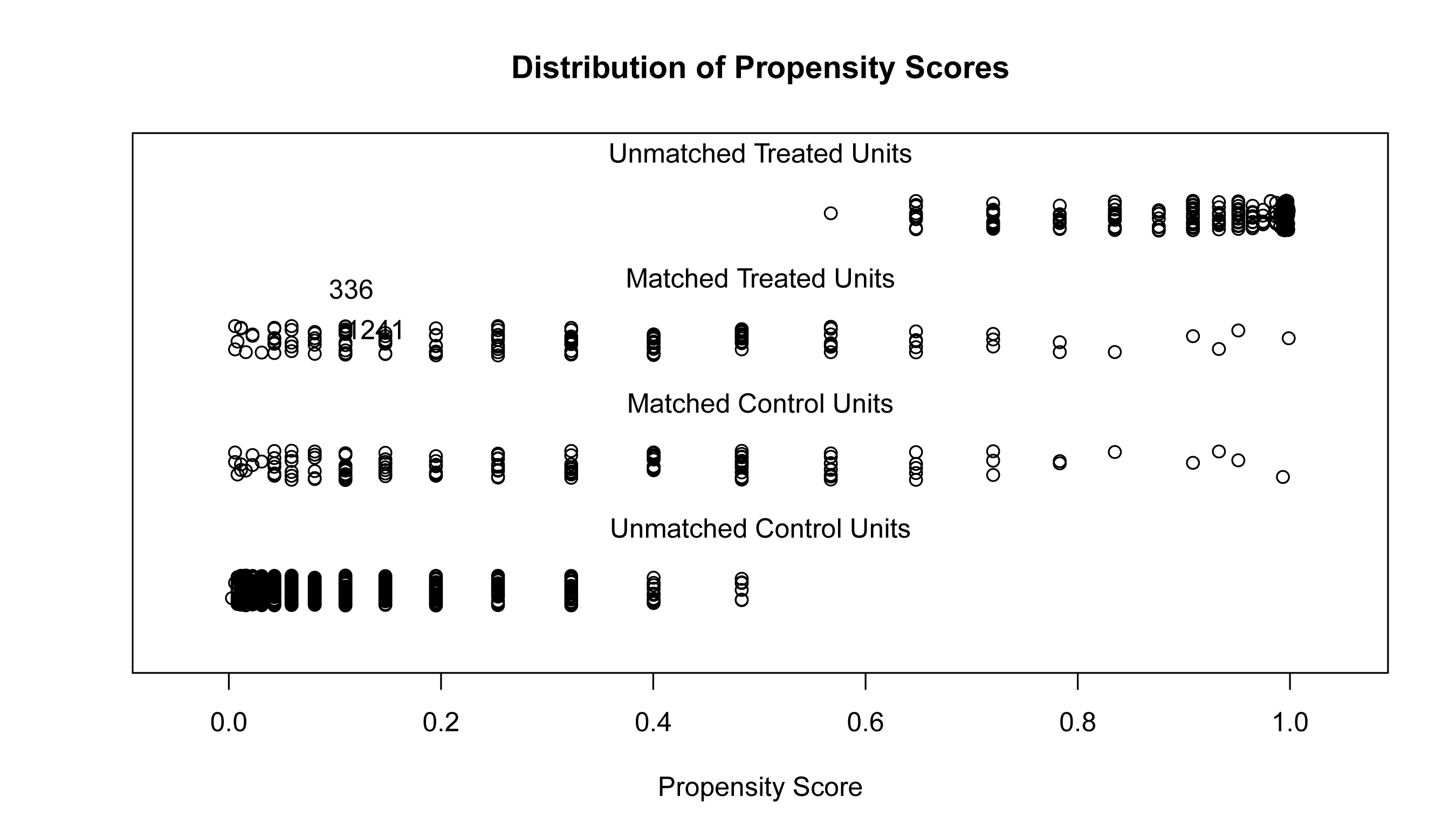

Supplement: Supplementary file 1 [file DataSheet_1.zip › Supplementary/Figue s2.tif]

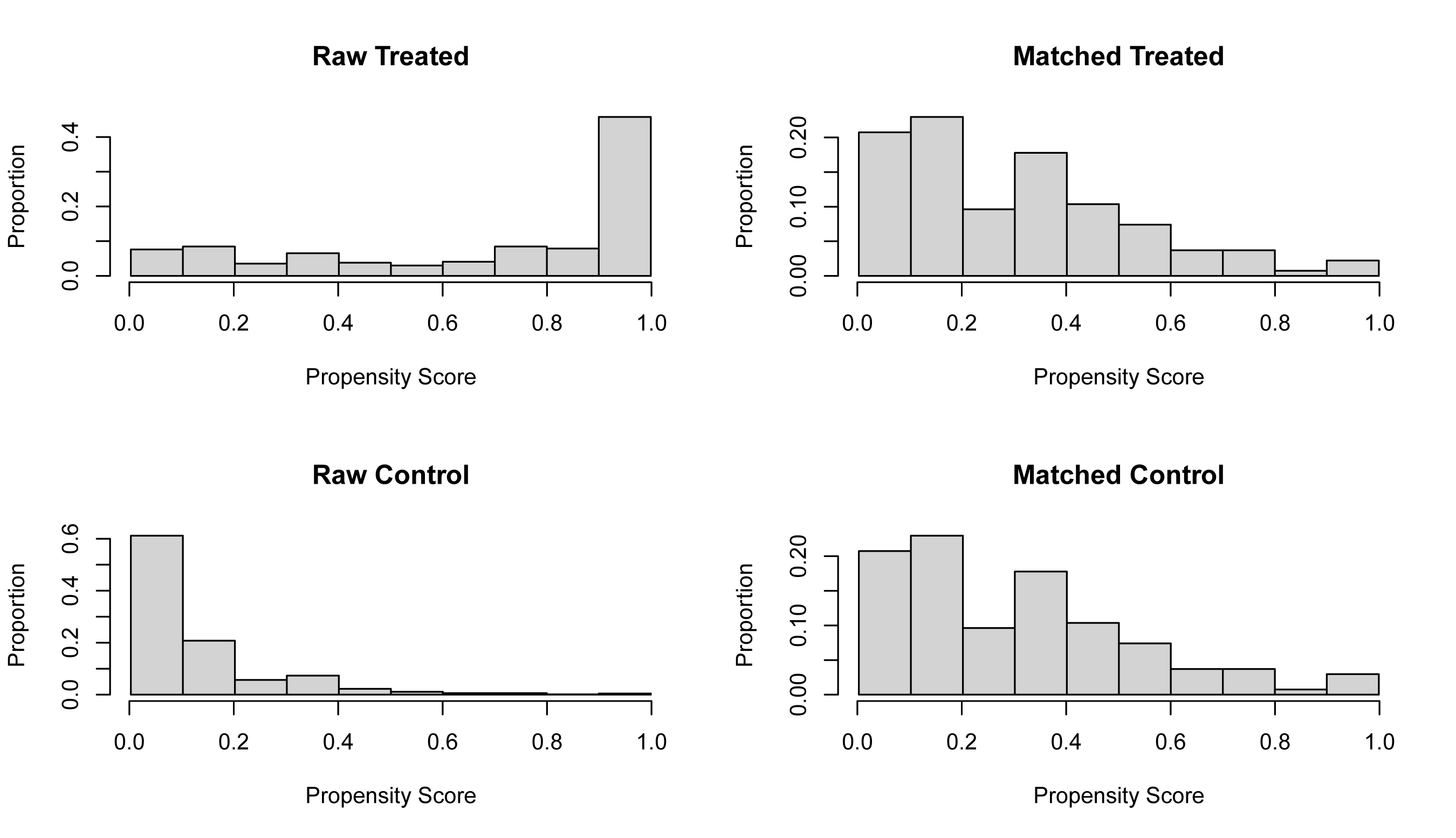

Supplement: Supplementary file 1 [file DataSheet_1.zip › Supplementary/Figue s3.tif]
